# Editorial: Insights in rhinology: 2021/22

**DOI:** 10.3389/falgy.2023.1221497

**Published:** 2023-06-12

**Authors:** Peter Kenneth Smith, Glenis Kathleen Scadding

**Affiliations:** ^1^ School of Medicine, Griffith University, Southport, QLD, Australia; ^2^ Division of Infection and Immunity, Faculty of Medical Sciences, University College London, London, United Kingdom

**Keywords:** chronic rhinosinusitis, allergy, olfaction, quality of life, COVID, vaccination, nasal route, type 2 inflammation

**Editorial on the Research Topic** Insights in rhinology: 2021/22

## Introduction

Chronic rhinosinusitis (CRS) and allergies pose significant health burdens, affecting millions of people worldwide, yet are often underappreciated, misdiagnosed, and ineffectively treated, leading to personal misery and societal losses in productivity. Recent research has provided new insights into the pathophysiology, diagnosis, and treatment of these conditions. In this Editorial, we review the key findings of several articles in our Research Topic, addressing the clinical features, mechanisms, and management of CRS and allergies, as well as the implications for patient care.

## Chronic rhinosinusitis

### Olfactory dysfunction in CRS

Smell has been described as the “Cinderella of the senses” since its importance to life is underappreciated until it is lost. Olfactory dysfunction is a prevalent and debilitating symptom of CRS, significantly impacting patients' quality of life and impairing their safety. Macchi et al. conducted a multicentric study examining the prevalence and severity of olfactory dysfunction in patients with CRS. The authors emphasized the importance of assessing olfactory function during diagnosis and treatment and suggested using standardized olfactory tests to better understand the impact of CRS on the sense of smell and the response to various therapies.

Lin and Yeh reviewed the current literature on the clinical features, mechanisms, and management of olfactory dysfunction secondary to CRS. The authors discussed the potential role of inflammatory mediators, oxidative stress, and neurodegenerative processes in its development and highlighted the need for further research to improve both diagnosis and treatment of olfactory dysfunction in patients with CRS.

### Uncontrolled disease in CRS

Viskens et al. explored various factors contributing to uncontrolled CRS and the challenges associated with effective management. The authors emphasized the importance of addressing the multifactorial nature of CRS, including the role of comorbidities, environmental factors, and patient adherence to treatment. Personalized treatment strategies and multidisciplinary care may lead to improved outcomes for patients with CRS.

## Inflammatory conditions and allergies

### Chronic type 2 inflammation of airways and skin

De Prins et al. published a white paper presenting an overview of European patient needs and suggestions regarding chronic type 2 inflammation of airways and skin. The authors highlighted the importance of patient-centred care and advocated for the development of standardized diagnostic tools and treatment protocols. The authors also emphasized the need for improved patient education and access to specialist care.

### Cat allergy

Hossenbaccus et al. conducted a literature review on cat allergy, emphasizing the need for controlled methodology in studying cat allergens and their impact on human health. The authors called for further research using standardized exposure protocols and assessment tools to advance our understanding of cat allergy and to inform evidence-based management strategies.

## Vaccine development and grand challenges

### Intranasal COVID-19 vaccine

Scadding proposed the development of an intranasal COVID-19 vaccine, highlighting the potential benefits of mucosal immunity in preventing SARS-CoV-2 infection. Current systemic vaccines reduce morbidity and mortality but have little effect on disease transmission. Intranasal vaccines may be more effective in this respect since they invoke mucosal immunity. The intranasal application of drugs, allergens, and vaccines represents an exciting new direction in rhinology research.

### Grand challenges in rhinology

Scadding also outlined the grand challenges in rhinology, providing a comprehensive overview of the most pressing issues in the field, including the need for improved diagnostics, personalized treatment approaches, and a better understanding of the genetic and environmental factors contributing to rhinologic conditions. The author also emphasized the importance of interdisciplinary collaboration, patient-centred care, and proper investigation of novel therapeutic strategies.

## Conclusion

The articles reviewed in this Editorial provide valuable insights into the current state of research and clinical practice in the fields of rhinology and allergy. By examining the complex interplay of factors contributing to CRS and allergies, these studies highlight the need for personalized and comprehensive care, as well as continued research to improve diagnostics, treatments, and patient outcomes. The grand challenges in rhinology, as outlined by Scadding, serve as a roadmap for the field, guiding researchers and clinicians toward innovative solutions for the most pressing issues in rhinology.

Addressing olfactory dysfunction in patients with CRS, as discussed by Macchi et al. and Lin and Yeh, is crucial for improving patient quality of life. The importance of understanding and addressing uncontrolled disease in patients with CRS, as highlighted by Viskens et al. underscores the need for personalized treatment approaches and a comprehensive understanding of the factors contributing to CRS.

De Prins et al. also emphasized the importance of patient-centred care and the development of standardized diagnostic tools and treatment protocols. This EUFOREA patient project is continuing with the development of checklists for patients to use when accessing care. Hossenbaccus et al.’s review of cat allergy also stresses the need for controlled methodology in studying allergens and their impact on human health.

Lastly, the proposal by Scadding for the development of an intranasal COVID-19 vaccine illustrates the potential for innovation and the application of rhinology research to broader public health concerns.

By addressing these various research areas and focusing on the grand challenges in rhinology, the field can continue to advance and improve the quality of care for individuals affected by CRS, allergies, and other diseases of the upper airway. Frontiers in Allergy/Rhinology wishes to contribute to this by providing convenient free open access to innovative thinking.

## Author's note

We used Chat GPT4 as an initial aide in writing this Editorial but were dismayed to find that it confabulated by producing references with wrong authors and failed to notice when there was only one author. The paranoia of one of us (GKS) has increased because this happened to both her articles.

